# Spatial Pattern and Scale Influence Invader Demographic Response to Simulated Precipitation Change in an Annual Grassland Community

**DOI:** 10.1371/journal.pone.0169328

**Published:** 2017-01-03

**Authors:** Meghan J. Skaer Thomason, Kevin J. Rice

**Affiliations:** Department of Plant Sciences, University of California, Davis, Davis, California, United States of America; Universidade de Vigo, SPAIN

## Abstract

It is important to predict which invasive species will benefit from future changes in climate, and thereby identify those invaders that need particular attention and prioritization of management efforts. Because establishment, persistence, and spread determine invasion success, this prediction requires detailed demographic information. Explicit study of the impact of pattern on demographic response is particularly important for species that are naturally patchy, such as the invasive grass, *Aegilops triuncialis*. In the northern California Coast Range, where climate change may increase or decrease mean annual rainfall, we conducted a field experiment to understand the interaction of climate change and local-scale patterning on the demography of *A*. *triuncialis*. We manipulated precipitation (reduced, ambient, or augmented), seed density, and seeding pattern. Demographic and environmental data were collected for three years following initial seeding. Pattern and scale figure prominently in the demographic response of *A*. *triuncialis* to precipitation manipulation. Pattern interacts with precipitation and seeding density in its influence on per-plant seed output. Although per-plot seed production was highest when seeds were not aggregated, per-plant seed output was higher in aggregated patches. Results suggest aggregation of invasive *A*. *triuncialis* reduces the detrimental impact of interspecific competition in its invaded community, and that interspecific competition *per se* has a stronger impact than intraspecific competition.

## Introduction

Spatial patterns are a crucial aspect of understanding invasion mechanisms [1,2]. Spatial aggregation or “patchiness” can have large impacts on competitive balance in a community [1] and can promote coexistence or competitive exclusion [3]. Causes of spatial pattern can be exogenous (i.e. arising from factors not due to plant species characteristics), such as soil pattern, structure, or nutrient distribution. Spatial pattern can also be generated by endogenous factors such as propagule dispersal characteristics or plant-plant facilitation or inhibition. Spatial patterns resulting from plant characteristics such as seed dispersal distance have been shown to influence community composition and alter competition between native and exotic species [4].

Climate change impacts on spatial pattern have been investigated in some systems (e.g. arid ecosystems [5]), particularly in the context of vegetation type transitions [5,6]. These studies have investigated plant distributions and the impact that local facilitation has on threshold transitions and types of pattern [5]. At a larger scale, vegetation pattern can influence the outcome of rainfall-land feedbacks [6] and ultimately influence drought severity or temporal rainfall distribution. For invasive plants, the details of the interaction of spatial pattern, such as aggregation, with plant demographic response to climate change has not been well investigated. Climate change and spatial pattern might interact to alter invasive species population dynamics. For example, spatial pattern may influence the relative impact of intra- vs. interspecific competition in invasive species for limiting resources (e.g., soil moisture) that may be influenced by predicted climate change (e.g., reductions or increases in annual rainfall).

Because many species distributions are highly patchy, models have recently focused on patchiness in understanding everything from interactions of the individual plant neighborhood to community and landscape dynamics [2]. For example, a ‘cellular automaton’ model of competitive coexistence [1] assessed neighborhood interactions, while other models address larger scale processes that structure population-level dynamics [7] or maintain species diversity [8]. Many of these multi-species neighborhood models are based on Markov chain theory, and thus predict the probabilities of whether a patch will remain the same, or transition to another state. However, none of these models are able to accurately estimate competitive interactions in monospecific patches. In these patches, we know conspecific interactions can be high relative to interspecific interactions, but it is difficult to estimate *per capita* competitive effects [9,10]. As a result, it is difficult to predict the likelihood of spread from one patch to another. In the case of invasive species, especially annual species, this likelihood of spread is highly dependent on seed production. For this reason, it is important to investigate experimentally the impact of increasing intraspecific interactions [3] on reproductive rates in patchy invasive species.

Population demography can also be an invaluable tool in evaluating potential evolutionary response to climate change. Phenotypic plasticity is reflected in trait variability [11], and variability in fitness traits is a critical piece of information in understanding the potential response of a population to selection [12]. For example, studying relative variation in seed number produced per individual (i.e. the reproductive hierarchy of a population) yields a measure of the “opportunity for selection” [13,14]. Although there is a need for exploring evolutionary potential in invasive species to improve invasibility models [15], the study of rapid evolution is not always practical or prioritized [15]. Thus, one resolution of the logistical limitations of these eco-evolutionary studies is to use demographic approaches to estimate reproductive hierarchies and thus the evolutionary potential of an invader to adapt to climate change.

The floristic province of California is expected to experience altered precipitation under most climate change scenarios [16]. This floristic province is a diversity hotspot where biological invasion and climate change may have synergistic effects on the loss of biodiversity and ecosystem services resulting from invasive species. Increasing nitrogen deposition, for example, may accelerate the rate of spread of some invaders or favor a specific suite of invasive plants [17]. Although valley grasslands within this province are one of the most heavily invaded plant communities on the planet [18], it is still vulnerable to additional negative impacts of invasions [19], including degradation of forage and habitat for native wildlife and depletion of soil nutrients and water resources [20]. A general loss of productivity in California’s grasslands as a result of large-scale exotic weed invasions has already been documented [20,21].

*Aegilops triuncialis* (barb goatgrass) is one of the most problematic weeds in California annual grasslands. *A*. *triuncialis* has a locally patchy distribution that has been observed by ecologists [22] and land managers [23,24]. One reason this pattern emerges may be due to improved performance when *A*. *triuncialis* individuals experience a higher proportion of intraspecific interaction versus interspecific interaction. Thus, high density aggregations create a less competitive environment than sparse individuals surrounded by heterospecifics [3,25]. Other reasons for this invasion pattern might be limited seed dispersal distance, or expansion since time of introduction from a central locus [26].

Historically, native perennial grasses, active during the late spring or early summer (“late season” in a Mediterranean climate) would have been dominant in many locations [27], but winter annual grasses have largely displaced them over the last two centuries [18,28]. This loss of late-phenology species is likely an additional boon for *A*. *triuncialis* since the invader may take advantage of late-season water resources not being used by the winter annual community because of its extended growing season (2–4 weeks beyond most winter annual species [29–31]). Further shifts in timing or magnitude of resource availability (caused by climate change or biotically-driven) may alter the competitive balance in California grasslands in favor of late-season invaders like *A*. *triuncialis* [32]. Therefore, we aim to understand how climate change will impact the population dynamics of *A*. *triuncialis* and whether distribution pattern may modulate competitive interactions. Specifically, we established a field experiment manipulating seasonal precipitation and *A*. *triuncialis* seeding patterns, and measured the demographic response of *A*. *triuncialis* to the interaction of simulated climate change and conspecific spatial patterns.

## Materials and Methods

### Study System

We established a three-year field experiment at the University of California Hopland Research and Extension Center (Mendocino County, CA; 39°00'58” N, 123°03'47” W). The study location was at 880 m elevation. Soils in the experimental area are deep and well-drained, with clay content ranging from 12 to 50% [33]. The study area soils are characterized as mixed Ultic Argixerolls and Typic Haploxeralts. Soil surface slope in the study area ranged from 5–20%. Directional aspect varied from northwest to southwest.

**Fig 1:**
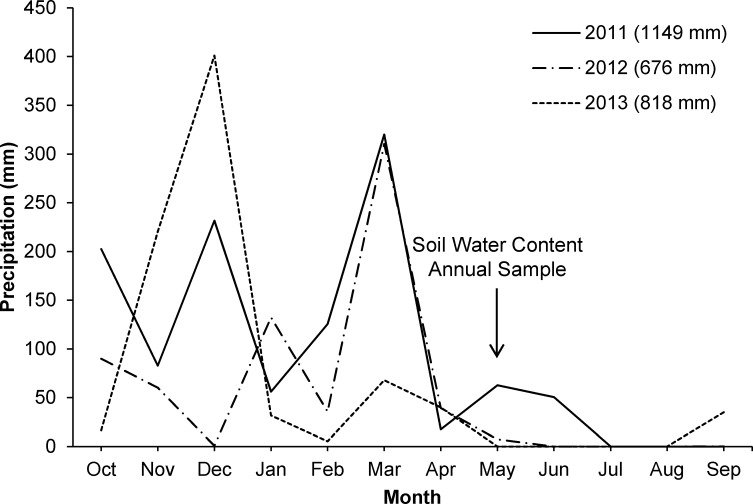
Annual precipitation (mm) during the study period (water-year from Oct 1 through Sep 30) near the study area (Ukiah, Mendocino County, California, USA; California Data Exchange Center). Precipitation totals for each year of the study are indicated in the figure key. Approximate timing of annual soil water content (%) sampling indicated with an arrow.

Climate in California’s annual grasslands is Mediterranean, with hot, dry summers and mild, wet winters. The study period fell within an unusual drought (2012–2014) in California [34]. Rainfall during the study period ranged from 676 mm to 1149 mm annually (Fig 1), measured Oct 1 to Sep 30 (water-year [35]). Of the three years of this study, the first year was the wettest water-year, and the second year was the driest. The third year of the study (2013) was characterized by an unusually dry spring (Jan-Jun) with only 17% of the year’s total precipitation falling during this period, compared to an average of 61% [36]. Spring rainfall during study years one and two (2011 and 2012) followed a more typical pattern, with heaviest precipitation falling during March (Fig 1).

Typically, the active growing season for annual grasslands in central and north-central California ends in late April or early May [20,28]. Precipitation in May and June of 2011 likely extended the growing season by up to a month because monthly totals were 1.5 and 5.5 times greater, respectively, than the 60-year mean [36]. The general lack of precipitation in spring of 2013, especially during May and June, likely resulted in an abbreviated growing season (up to a month reduction).

### Experimental Design

We used a split plot, factorial design that allowed exploration of the interactive effects of simulated climate change and spatial pattern on demographic responses of *A*. *triuncialis*. Six blocks, measuring 2 m × 6 m, were each divided into three main plots (2 m × 2 m) that were randomly assigned one of three levels of precipitation treatment: high, ambient, or reduced. Our plot layout allowed for 40 cm buffers to account for the possibility of edge effects between main plots. To reduce rainfall, semi-permeable “rain shelters” were installed in the first water year (January 2011) and reduced precipitation by approximately 25%. Water collected from these shelters was applied to the high-precipitation treatment areas within 24 hr of each rain event. Ambient plots received only incident rainfall. The range of rainfall manipulation reflects the range of predicted climate change [16,37]. The 25% reduction or augmentation in rainfall was relative to each year’s actual rainfall.

We constructed rain shelters modeled after a well-tested design [38] using a synthetic (PVC pipe) frame that supported six sloping ‘U’-shaped channels of clear polycarbonate. The polycarbonate channels (Rooflite, Co-Ex Corporation) allowed more than 90% of photosynthetically active radiation to penetrate; this small reduction in PPFD caused by the shelters is not likely to significantly reduce growth [38]. Each channel was 11 cm wide and spanned the 2 m frame at a 20° slope. The slope allowed efficient water runoff and caused minimum interference with light. Runoff from these channels was collected in a larger channel that directed intercepted water to a covered PVC container. Potential unintended impacts of water augmentation might include physical disturbance or changes to the oxygen state of the soil. To reduce the possibility of physical disturbance, water was lightly applied using a watering can fitted with a shower nozzle that minimized physical impact to the soil. Due to the well-drained rocky soil texture and sloping soil surfaces of the experimental plots, pooling of water due to treatment application that might lead to anoxic soil conditions was largely prevented, although water retention capacity and oxygen state of the soils is expected to vary across the experimental area.

**Fig 2:**
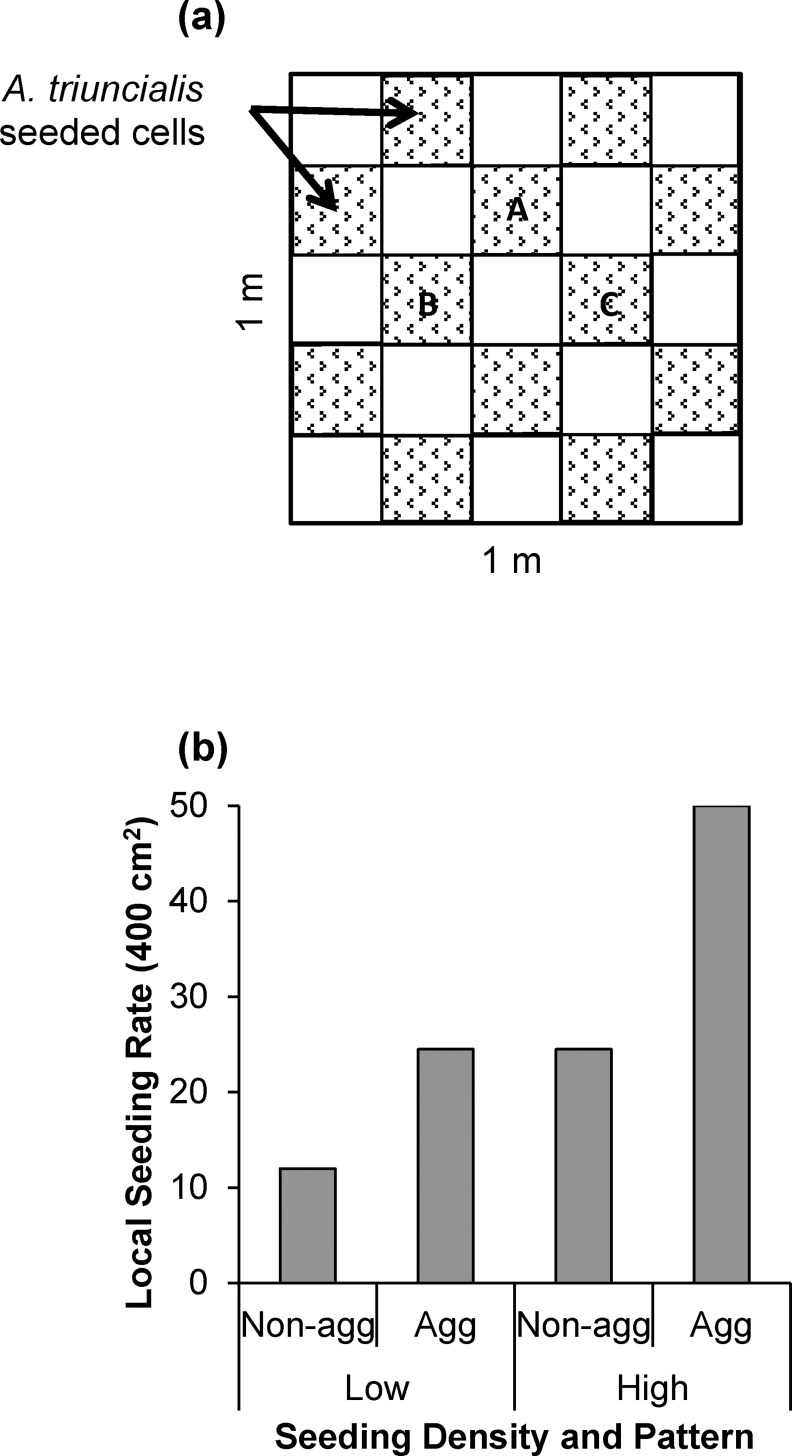
Diagram of experimental seeding pattern and density. A) The aggregated (checkerboard) pattern seed distribution treatment in a 1 m × 1 m subplot. Each stippled cell represents seeding locations of *Aegilops triuncialis*. Cells labeled A-C represent sub-sample locations. B) Local densities in each treatment cell for low and high density seeding rates applied without pattern (“Non-agg”) or with pattern (“Agg”).

Plot areas were selected in uninvaded (<5% cover of *A*. *triuncialis*) open grassland with minimal shading from tree canopy. Within each precipitation treatment main plot, four 1 m × 1 m subplots were established and randomly assigned a factorial combination of seed density (high, ~600 seeds/subplot; low, ~300 seeds/subplot) and seed pattern (patterned or evenly distributed). The patterned (“aggregated”) seed distribution indicates initial seed distribution in a ‘checkerboard’ pattern (Fig 2A). The evenly distributed seed treatment (“non-aggregated”) achieved a consistent density throughout a given subplot. Actual seed density calculated in a 20 cm × 20 cm treatment cell (Fig 2A) increased when aggregated (Fig 2B). The resultant aggregated cell density in low density, aggregated subplots matches that of the non-aggregated high density subplots (Fig 2B). This aspect of the experimental design allowed direct comparison of the effect of pattern exclusive of differences in density at the cell-scale. In both seeding pattern treatments, seed was applied over the existing, established community and resident seed bank. Seed was applied in October 2010 immediately preceding a large rain event. At the time of seeding, we trimmed and removed the dead, standing biomass in each subplot, applied seed, and reapplied the biomass in an even layer. This procedure allowed us to place *A*. *triuncialis* seeds below the litter layer and at the soil surface. *A*. *triuncialis* seed spikes would naturally penetrate the litter layer due to their weight and shape.

### Experimental Spatial Scales

The focal spatial scales of this experiment ranged in size from 1 cm^2^ to 10^4^ cm^2^. The smallest scale focused on individual plant responses that included per-plant reproductive rate. A mid-level spatial scale compiles individual plant responses within the 20 cm × 20 cm treatment cells (Fig 2A) into a ‘local response’ that includes cumulative reproduction and net reproductive rate. Finally, at the largest scale (1 m^2^), we observed the cumulative ‘subplot response’ that accounts for spatial heterogeneity in reproductive measures.

### Sampling and Measurements

To assess treatment effects on demographic performance, we imposed a cell grid of 20-cm squares on each 1 m × 1 m subplot (identical to the seeding pattern grid; Fig 2), and chose three cells (“sub-sample cells”) in each subplot for our measurements; these cells were centrally located in order to avoid potential edge effects of neighboring treatments. We evaluated demographic response of individual plants, measured propagule output at different spatial scales (local, subplot), and then calculated net reproductive rate. To do this, we calculated seed density in each year, and these data were used to calculate net reproductive rate R_0,_ where R_0_ = N_t+1_/N_t_.

During the peak growth period of each year when resident species and *A*. *triuncialis* were both using soil water resources at a rapid rate (29 April 2011, 5 May 2012, and 27 April 2013; Fig 1), we measured volumetric water content with a time domain reflectometry soil probe (Field Scout TDR Soil Moisture Meter, Spectrum Technologies, Inc. [39]). Soil water content was measured once in each of the sub-sample cells at 15 cm depth. Due to the rocky nature of the field soil, measurements within a sub-sample cell were often repeated until an accurate (non-zero) reading was achieved.

At the end of the growing season, we measured *A*. *triuncialis* adult density, and for a haphazard selection of four adult plants in each of the three sub-sample cells, we also measured the number of spikelets produced. From these numbers, and accounting for our seeding and sampling design, we calculated total spikelet output per subplot. Enumeration of spikelets provides an accurate index of reproductive output in *A*. *triuncialis* because each spikelet consistently contains a set of paired florets [40]. As a measure of population growth, seed density in each year was used to calculate net reproductive rate per generation (R_0_) where R_0_ = N_t+1_/N_t_.

### Statistical Analyses

A repeated measure multivariate general linear model (MANOVA) was used to determine whether our treatments had an effect on the demographic variables (spikelets/plant, plant density, and seeds/subplot). Soil water content was analyzed separately. We used Pillai’s Trace to evaluate significance in the MANOVA. If the MANOVA was found to meet the significance criteria (p < 0.05), we used a ‘protected’ ANOVA protocol [41] to test each demographic variable in individual repeated measure general linear models (GLMs). All multivariate and univariate GLMs tested the same treatment factors: year, water treatment, seeding pattern, and seeding density. Non-repeated factors in univariate models had 45 degrees of freedom and were evaluated using Type III sums of squares. Repeated factors and multivariate models had varying degrees of freedom depending on the number of response variables included in the trait group and the factor. All analyses were performed in SAS (Version 9.3, SAS Institute Inc., Cary, NC, 2010) using the general linear model procedure.

## Results

### Water: Rainfall Manipulation and Interannual Variation

The rainfall manipulation treatment affected volumetric soil water content (F_2,45_ = 5.78, p = 0.0214); soil water in plots exposed to ambient rainfall was 6.9%. Comparatively, soil in sheltered plots had 4% less soil water (6.6% soil water content), and augmented plots had 7% higher soil water (7.4% soil water content). Mean soil water content in ambient plots ranged from 2.7 to 10.5% across study years, compared to 53% measured at field capacity [42]. The lowest water content averaged across all rainfall treatments was recorded in spring of 2013. The preceding water year (2012–2013) did not have the lowest total rainfall, but had the least rainfall in the later spring months (March-June; Fig 1). There were interactions among year, block, and rainfall manipulation for all of the variables analyzed (Table 1). Soil water content responded to the interaction of year and block (F_10,90_ = 9.27, p = 0.0001); this interaction suggests that soil moisture response to the large variation in rainfall among years was also influenced by spatial variation among blocks in edaphic factors and aspect (Table 2).

**Table 1:** Results from a MANOVA, followed by protected repeated measure general linear models for five demographic response variables in *Aegilops triuncialis*: number of spikelets per plant (A), coefficient of variation for spikelets per plant (B), number of spikelets produced per cell (C), number of spikelets produced per sub-plot (D), net population growth rate (E).

Model Effect	F	DF	*p*
**MANOVA**	4162.63	5,39	**0.0001**
**A. Spikelets per Plant**			
**Block**	18.55	5	**0.0001**
**Precipitation**	18.81	2	**0.0004**
Density	2.13	1	0.1514
**Pattern**	4.64	1	**0.0367**
Block*Precipitation	1.07	10	0.4021
Precipitation*Density	2.89	2	0.0657
Precipitation*Pattern	0.01	2	0.9884
Density*Pattern	1.06	1	0.3090
Precipitation*Density*Pattern	4.18	2	**0.0217**
**Year**	46.84	2	**0.0001**
Year*Block	5.47	10	**0.0001**
Year*Precipitation	0.61	4	0.6613
Year*Density	1.48	2	0.2385
Year*Pattern	1.12	2	0.3348
Year*Block*Precipitation	2.00	20	**0.0146**
Year*Precipitation*Density	1.38	4	0.2476
Year*Precipitation*Pattern	1.08	4	0.3729
Year*Density*Pattern	2.32	2	0.1103
Year*Precipitation*Density*Pattern	0.79	4	0.5316
**B. CV (Spikelets per Plant)**			
**Block**	13.85	5	**0.0001**
**Precipitation**	13.51	2	**0.0014**
Density	2.09	1	0.1548
Pattern	1.38	1	0.2460
Block*Precipitation	0.63	10	0.7845
Precipitation*Density	1.52	2	0.2300
Precipitation*Pattern	0.25	2	0.7776
Density*Pattern	0.22	1	0.6445
Precipitation*Density*Pattern	2.09	2	0.1358
**Year**	14.54	2	**0.0001**
Year*Block	3.17	10	**0.0016**
Year*Precipitation	1.8	4	0.1693
Year*Density	3.64	2	**0.0344**
Year*Pattern	1.33	2	0.2762
Year*Block*Precipitation	1.05	20	0.4112
Year*Precipitation*Density	0.89	4	0.4712
Year*Precipitation*Pattern	0.08	4	0.9893
Year*Density*Pattern	2.84	2	0.0691
Year*Precipitation*Density*Pattern	0.48	4	0.7473
**C. Spikelets per Cell**			
**Block**	15.37	5	**0.0001**
**Precipitation**	4.45	2	**0.0416**
Density	0.10	1	0.7525
Pattern	3.62	1	0.0634
Block*Precipitation	4.72	10	**0.0001**
Precipitation*Density	0.49	2	0.6167
Precipitation*Pattern	2.12	2	0.1321
Density*Pattern	6.12	1	**0.0172**
Precipitation*Density*Pattern	5.65	2	**0.0065**
**Year**	63.71	2	**0.0001**
Year*Block	5.26	10	**0.0001**
Year*Precipitation	2.38	4	0.0864
Year*Density	0.07	2	0.9329
Year*Pattern	2.04	2	0.1424
Year*Block*Precipitation	2.15	20	**0.0079**
Year*Precipitation*Density	2.37	4	0.0588
Year*Precipitation*Pattern	1.20	4	0.3148
Year*Density*Pattern	3.31	2	**0.0459**
Year*Precipitation*Density*Pattern	2.77	4	**0.0321**
**D. Spikelets per Subplot**			
**Block**	8.43	5	**0.0001**
**Precipitation**	4.31	2	**0.0446**
Density	0.45	1	0.5065
**Pattern**	13.92	1	**0.0005**
Block*Precipitation	2.75	10	**0.0099**
Precipitation*Density	2.37	2	0.1050
Precipitation*Pattern	0.08	2	0.9246
Density*Pattern	3.52	1	0.0672
Precipitation*Density*Pattern	6.58	2	**0.0031**
**Year**	53.39	2	**0.0001**
Year*Block	5.24	10	**0.0001**
Year*Precipitation	1.64	4	0.2023
Year*Density	0.37	2	0.6907
Year*Pattern	3.57	2	**0.0365**
Year*Block*Precipitation	1.67	20	0.0533
Year*Precipitation*Density	0.90	4	0.4646
Year*Precipitation*Pattern	1.33	4	0.2632
Year*Density*Pattern	3.20	2	0.0504
Year*Precipitation*Density*Pattern	1.93	4	0.1129
**E. Net Reproductive Rate**			
Block	2.05	5	0.0901
Precipitation	1.36	2	0.2683
Density	0.34	1	0.5622
Pattern	1.39	1	0.2456
Block*Precipitation	0.43	10	0.9218
Precipitation*Density	0.17	2	0.8431
Precipitation*Pattern	1.10	2	0.3405
Density*Pattern	1.29	1	0.2619
Precipitation*Density*Pattern	0.08	2	0.9278
**Year**	35.58	2	**0.0001**
Year*Block	2.12	10	**0.0308**
Year*Precipitation	2.27	4	0.0978
Year*Density	2.07	2	0.1394
Year*Pattern	3.55	2	**0.0376**
Year*Block*Precipitation	1.11	20	0.3587
Year*Precipitation*Density	0.37	4	0.8308
Year*Precipitation*Pattern	1.13	4	0.3501
Year*Density*Pattern	1.45	2	0.2469
Year*Precipitation*Density*Pattern	0.87	4	0.4867

**Table 2:** Results from a repeated measure GLM for soil water content (%).

Model Effect	F	DF	*p*
**Block**	147.54	5,45	**0.0001**
**Precipitation**	5.78	2,45	**0.0214**
Density	0.02	1,45	0.8798
Pattern	0.22	1,45	0.6440
Block*Precipitation	0.73	10,45	0.6934
Precipitation*Density	0.71	2,45	0.4984
Precipitation*Pattern	0.82	2,45	0.4482
Density*Pattern	1.18	1,45	0.2834
Precipitation*Density*Pattern	0.19	2,45	0.8298
**Year**	1000.24	2,44	**0.0001**
Year*Block	9.27	10,90	**0.0001**
Year*Precipitation	1.60	4,20	0.2125
Year*Density	0.64	2,44	0.5332
Year*Pattern	0.70	2,44	0.5043
Year*Block*Precipitation	1.37	20,90	0.1614
Year*Precipitation*Density	0.68	4,90	0.6104
Year*Precipitation*Pattern	1.70	4,90	0.1569
Year*Density*Pattern	1.49	2,44	0.2374
Year*Precipitation*Density*Pattern	1.04	4,90	0.3928

### Demographic Response

**Fig 3:**
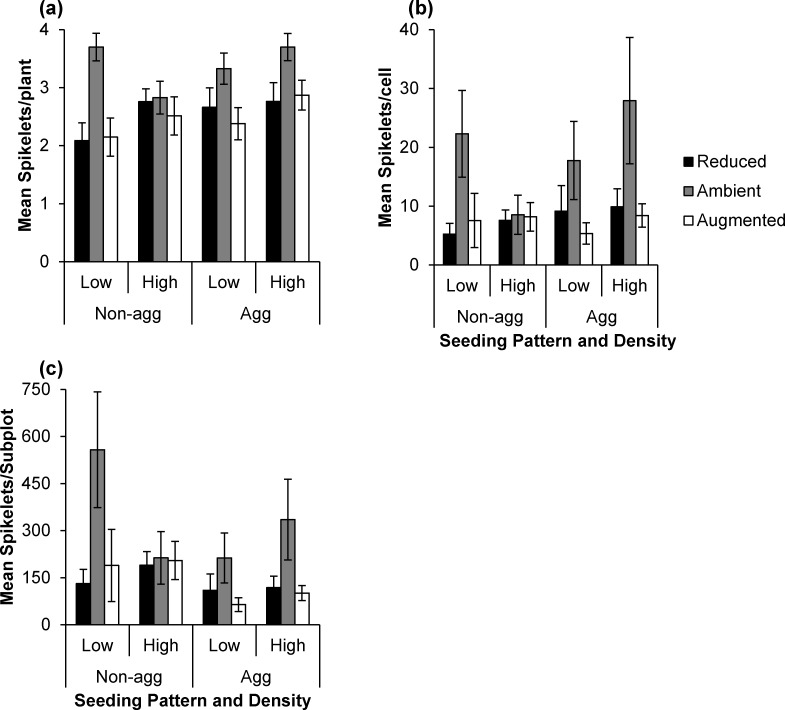
Demographic response of *Aegilops triuncialis*. Mean (±SE) A) spikelet number per adult individual plant, B) spikelet number per treatment cell, and C) spikelet number per treatment sub-plot grouped by precipitation treatment, seeding density (low or high), and seeding pattern (non-aggregated or aggregated).

A multivariate test of demographic variables was significant (F_5,39_ = 4162.63, p = 0.0001; Table 1) and allowed us to proceed with protected univariate tests on each variable: spikelets per plant, spikelets per cell, spikelets per plot, CV in spikelets per plant, and net reproductive rate. In general, both high and low rainfall decreased seed production in all spatial and density treatments with the exception of the high density, non-aggregated seeding treatment (Fig 3). The lack of response to precipitation in the high density, non-aggregated seeding treatment for spikelets per plant, spikelets per cell, and spikelets per plot is reflected in the significant three-way interaction of precipitation, density, and pattern for each of these seed response variables.

#### Individual plant response

The interaction of rainfall manipulation, aggregation pattern, and seeding density significantly affected the number of spikelets per *A*. *triuncialis* adult plant (Table 1A). The low density, non-aggregated distribution seeding treatment had the lowest per-plant reproductive output (Fig 3A), and the reduction due to each rainfall treatment for these plants was more than 40%. Specifically, augmented rainfall led to 26% fewer spikelets per individual plant, and reduced rainfall, 23% fewer. Overall, plants in the aggregated treatment had a 10% higher per-plant reproductive output.

**Fig 4:**
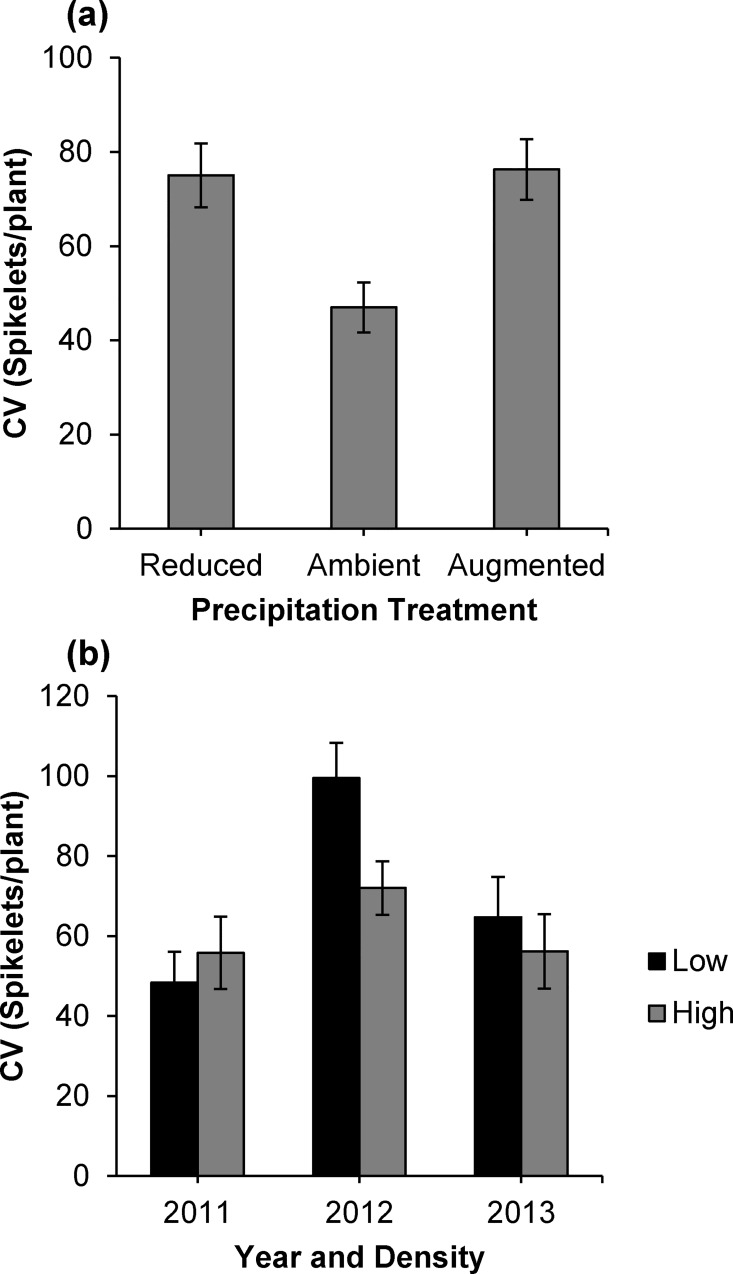
Mean (±SE) coefficient of variation (CV) in spikelet number per *Aegilops triuncialis* adult individual plant. Response grouped by A) precipitation treatment, and B) year and seeding density.

Analysis of relative variation (CV) in spikelet production per plant revealed a main effect of rainfall manipulation. Compared to the ambient rainfall treatment, relative variation in spikelet production was 60% higher under the decreased rainfall treatment and 62% higher under the augmented rainfall treatment (Fig 4A). There was also an interactive effect of year and seeding density on relative variation in spikelet production (Table 1B). Overall, relative variation was 65% higher in 2012 than 2011, and 42% higher than 2013. Low-density plots in 2012 exhibited a particularly high relative variation in spikelet production that was 38% higher than their high-density counterparts in the same year (Fig 4B). In fact, 2012 and 2013 both followed this trend for increased relative variation in low-density plots, but there was an opposite trend in 2011.

#### Local-scale response

Estimated spikelet production at the local (20 cm × 20 cm treatment cell) scale responded to an interaction of rainfall, seeding pattern, and seeding density (Table 1C). We found the lowest number of spikelets per cell in the low density non-aggregated seeding treatment, where reduced rainfall led to a 76% reduction in spikelets per cell, and augmented rainfall led to a 66% reduction (Fig 3B). The magnitude of this reduction is similar to reductions in the high density aggregated treatment. Similar to the results for spikelets per plant, local-scale spikelet numbers reveal a strong negative response to the altered rainfall treatments (50% reduction on average), with the exception of the high density-even seed distribution treatment.

**Fig 5:**
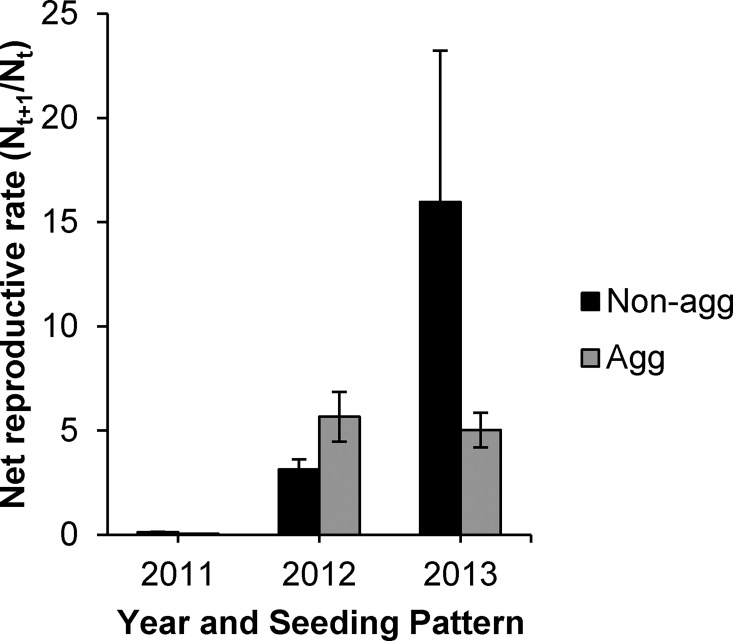
Mean (±SE) estimated net reproductive rate of *Aegilops triuncialis* grouped by year and seeding pattern (non-aggregated or aggregated).

There was a significant interactive effect of year and seeding pattern (Table 1E) on net reproductive rate; however, this is the only variable not affected by rainfall manipulation. Aggregated seeding led to a stable net reproductive rate across 2012 and 2013; however, the plants in non-aggregated seeding treatment displayed a wide variation in net population growth rate across years. In 2012, plants in non-aggregated seeding treatment had a 44% lower net reproductive rate (Fig 5) while in 2013 plants within non-aggregated treatments exhibited a 68% higher net reproductive rate. Mean net reproductive rate overall was very low in the first year due to low germination and survival of experimentally applied seeds. However, in subsequent years, the plots supported a greater proportion of propagules to reproductive stage; mean net reproductive rate exhibited a greater than 4-fold increase in 2012 and a greater than 10-fold increase in 2013 (Fig 5).

#### Subplot-scale response

The interaction of rainfall manipulation, seeding density, and seeding pattern also affected subplot-scale spikelet production (Table 1D). The strong negative response of per-plant reproduction under both rainfall treatments in the low density non-aggregated treatment was magnified at this scale by the total number of treated cells in each subplot (25 for non-aggregated, 12 for aggregated; see Methods). In the high density-aggregated seed treatments, per-subplot spikelet production under both altered rainfall treatments was also low when compared to the ambient rainfall treatment (Fig 3C). Over three years, the relative difference between spikelet production in the low density, non-aggregated subplots and high density, aggregated subplots became smaller (Table 1D), decreasing from 58% to 33%.

## Discussion

For populations of *A*. *triuncialis* invading California grasslands, we suggest that aggregation reduces the negative impact of interspecific competition that, in turn, may have a stronger negative impact than intraspecific competition. We increased the amount of intraspecific competition in *A*. *triuncialis* to measure the impact of this potential difference in competition strength of conspecific and heterospecific interactions. Weak interspecific competitors may persist in aggregated, conspecific clumps because of fewer negative interactions with heterospecifics [3,25,43]. Reduced dispersal in *A*. *triuncialis* because of large, heavy spikes may also contribute to increased aggregation, and thus increased spatial competitive segregation. Limited dispersal can lead to evolutionary development of reduced competition among relatives [44] because success depends on their ability to thrive near their genetic siblings. This may be especially important for self-pollinating species, such as *A*. *triuncialis*, that exhibit very strong genetic similarity among siblings [45].

Our evaluation of relative strengths of interaction is not only important in understanding the spatial demography of *A*. *triuncialis*, but also has implications for other invasive species, and coexistence theory in general. Near monospecific, aggregated stands of invasive plant species are a very common phenomenon, and our research describes the demographic patterns underlying this aggregated spatial structure. Mechanisms of species coexistence have been reviewed and grouped into two categories: stabilizing or equalizing [46]. Stabilizing mechanisms are driven by degree of niche overlap between competing species and resource partitioning, and result in greater intraspecific competition compared to interspecific competition [47]. Equalizing mechanisms, in contrast, operate by minimizing differences in fitness to alter competitive outcomes between species in favor of the weaker competitor [46,47]. Of the mechanisms discussed in the review, aggregation is the only equalizing mechanism [46]. Initially, the idea of aggregation as a mechanism for coexistence was described [48], and subsequently modeled [1]. However, evidence for the aggregation mechanism is lacking in realistic field settings [46]. Clifford and Sudbury modeled species that were evenly matched [48], and found that aggregation was favored. Subsequent studies [3,25,43] have since shown that coexistence among species is favored by aggregation even when species are not evenly matched. A cellular automaton model [1] also indicated that aggregation leads to coexistence among unevenly matched species. The inferior competitor is the beneficiary of spatial competitive segregation in the context of plant coexistence. If we are correct in our hypothesis that *A*. *triuncialis* is a weak interspecific competitor, this fact could explain the persistence of aggregated distribution patterns of this invader within a matrix of stronger interspecific competitors.

We found a contrast between per-plant seed output and subplot-scale seed output. When adjusted mathematically for initial seeding pattern, per-subplot reproduction was surprisingly high in the aggregated treatments. Over three years, the data suggest per-plant seed output was rising, possibly indicating a delayed per-plant reproductive response of plants in aggregated treatments, although this was not supported statistically. These data do not include plants invading the unseeded cells, so this suggests that over a longer time scale, an aggregated stand of *A*. *triuncialis* might attain higher population growth rates and higher total spikelet production than an evenly distributed stand.

Net reproductive rate response to aggregated seeding treatment was relatively consistent across years. In contrast, the non-aggregated treatment showed considerable variation between 2012 and 2013. The 2012 water year was the driest in the study, and 2013 was characterized by a dry spring. This suggests that local aggregation may foster year-to-year stability in population level reproductive output. In desert systems, annual reproductive stability has been shown as an effective evolutionary response to highly variable environments ([49] and citations therein), thus leading to long-term population survival. However, non-aggregated populations, where *A*. *triuncialis* is experiencing more interspecific interactions, exhibit a perhaps more opportunistic response under stressful conditions. This opportunism may reflect the capacity of *A*. *triuncialis* individuals to respond to variation in the competitive effect of neighboring heterospecifics that, in turn, may respond more strongly to yearly variation in rainfall pattern. Because our study occurred during a period of drought, these differences in response among years likely reflect a range of responses to varying extremes of drought stress, rather than the full range of responses to yearly variation in precipitation.

Reproduction decreased in response to both rainfall treatments, indicating decreased reproductive output under either precipitation change scenario. Thus, depending on the trajectory of climate change in California grasslands, our results could indicate a few alternative outcomes. For example, if annual rainfall variability in California is exacerbated by climate change, aggregation would likely be favored at long time scales because of competitive segregation benefits [3,43] and the long-term population survival benefits attained through reproductive stability [49]. On the other hand, if climate change in California simply leads to decreased total rainfall (i.e. decreased soil moisture), the opportunistic increase of reproduction in non-aggregated plants could be favored if successful years compensate for unsuccessful years, although literature on population dynamics in variable environments suggests this is not likely [49]. It is likely we may see a combination of these changes in climate, with increasing variability and likelihood of unusual mid-season droughts, combined with decreasing total rainfall. Therefore, we may continue to see both distribution patterns perpetuated in the landscape. In fact, the ability of *A*. *triuncialis* to succeed in aggregated or non-aggregated distributions may be a critical bet hedging and risk management strategy, making this troublesome invader especially recalcitrant in the face of management.

Under both rainfall manipulation treatments, we measured increased relative variation in *per capita* reproductive rates that, in turn, indicates greater reproductive hierarchy. A more pronounced reproductive hierarchy reduces effective population size, and thus the chance for drift effects is greater and the effectiveness of selection is reduced [50]. One explanation for this unusual result is that the rainfall treatment was not effective at a biologically relevant scale and therefore results are ambiguous; however, small relative differences in soil water content can have meaningful impacts on growth under stressful conditions. Therefore, different mechanisms are likely at play in either precipitation treatment. It is possible that there is reduced survival in the low rainfall treatment, which would increase reproductive hierarchy (i.e. more zeros for seed output). The reduced opportunity for selection under the reduced rainfall manipulation is similar to results found for *A*. *triuncialis* in dry serpentine soils [12]. On the other hand, under increased rainfall there may be a greater proportion of very large individuals, which would also increase reproductive hierarchy within the experimental populations. Because our data collection is based on a sub-sample of the experimental populations and not a full census, we cannot distinguish between these demographic possibilities.

Overall, our results suggest some approaches for improving management of *A*. *triuncialis* in natural areas. Increasing the probability of interspecific interactions would likely be detrimental to *A*. *triuncialis* infestations. For example, fragmentation of *A*. *triuncialis* patches resulting from well-timed grazing by domestic range animals might slow population growth. Another option would be seed augmentation in a restoration context. This would be especially successful when seeding the strongest competitors of *A*. *triuncialis*. More research would be required to investigate the potential for native species candidates. Other studies have suggested management prioritization frameworks based on spatial pattern, such as targeting satellite individuals rather than large parent patches of invasive *Spartina* [7]. Our results indicate that neither high-density patches, with the potential for creating heavy propagule pressure in the long term, nor the opportunistic satellite individuals, should be ignored in the management context. It is important that short term trends in climate (year-to-year variability) as well as long term trends (increasing or decreasing annual totals) are considered when determining management priorities for invasive species such as *A*. *triuncialis*.
